# Author’s Reponse to Peer Reviews of “Insider Threats to the Military Health System: A Systematic Background Check of TRICARE West Providers”

**DOI:** 10.2196/57116

**Published:** 2024-04-09

**Authors:** David Bychkov

**Affiliations:** 1 UC Institute for Prediction Technology, University of California, Irvine, Orange, CA, United States

**Keywords:** TRICARE, health care fraud, Defense Health Agency, fraud, fraudulent, insurance, coverage, beneficiary, beneficiaries, background check, background checks, demographic, security clearance, FDA, Medicaid, Medicare, provider, provider referral, military, False Claims Act, HIPAA breach, OIG-LEIE, inspector general, misconduct, insider threat, information system, zero trust, data management, Food and Drug Administration, Health Insurance Portability and Accountability Act breach, Office of Inspector General's List of Excluded Individuals and Entities

*This is the authors’ response to peer-review reports for “Insider Threats to the Military Health System: A Systematic Background Check onf TRICARE West Providers.”*

## Round 1 Review

### Anonymous [1]

#### General Comments

*In general, the manuscript [2] is informative and includes a lot of information on health care providers who participate in TRICARE insurance. The study examines those who have received some sort of exclusion, sanction, or other reprimand based on health care fraud or harm. This study is timely and has practical implications for protecting patient care, particularly for those who are in a vulnerable position such as veterans or warfighters. I hope the following comments are taken as constructive criticism and interest in the overall improvement of the study. I appreciate the opportunity to review this study.*

*Below is a list of important fixes that I recommend considerable time be spent on and some minor fixes. In general, I think the key limitation of the study is that it can better state the significant contribution of the study. I understand the need for such a study, but as it stands, the study can further improve by spending more time on why and how health care providers land on exclusion lists such as the Office of Inspector General’s (OIG) List of Excluded Individuals and Entities (LEIE). Indeed, the study uses several databases that exclude physicians or provide reasons why a physician no longer participates in such programs, but the author can improve their justification for the study on why this is needed.*

*The second key limitation of the study is the Methods and Results section. In particular, this section needs improvement with clearer detail and justification on why the author had a selection criterion (vs examining all zip codes). In addition, the Results section can improve with better organization of the findings. As it reads, the results are a bit difficult to follow with all the zip codes laid out.*

*Last, the study could benefit from greater discussion on the implications of the study. At the moment, it pushes for more transparency, but the author could use their data more to discuss the impact of their findings. For example, why would publishing the National Provider Identification (NPI) numbers help patients? What do patients or the author want to gain from that transparency? How can this help future patients or hold physicians more accountable? The discussion loosely taps into the implications, but the study could really tease out this argument more.*

*Overall, the study was easy to follow and did provide some interesting content to consider. I think the study can better serve the public and has great implications! I would like to see these implications highlighted more so that the reader can really see the contribution the study makes.*

#### Specific Comments

##### Major Comments

*1. Introduction: Provide an explanation of what the OIG LEIE is for the reader. It is important to inform the reader that the OIG LEIE excludes participation in the program for various reasons—not just a quality-of-care issue. For example, the OIG LEIE also can exclude physicians on a financial offense matter. This helps the reader understand the gravity of the situation, particularly when the author discusses the increased risk of mortality and hospitalization of these patients. In short, I would like to see more development and background on how individuals are included in the LEIE to increase awareness for the reader.*

*a. For more information about LEIE and how physicians are placed on the list, please see the following: Burton B, Sun D, Jesilow P, Pontell HN. Two paths, one destination: a demographic portrait of physicians sanctioned by the federal government. J Health Hum Services Adm. 2022;45(3):142-180.*

Response: I have tried to add additional context about all the various lists, including the OIG LEIE. Ironically, I suspect we learned the most about TRICARE administrators from my article. Where they do enforce, there are exclusion-provider name matches.

*2. Methods: This section needs improvement. First, please provide more justifications and in-text citations to justify the methods used for the study. This will help strengthen the Methods section. As it reads now, there seem to be no prior studies listed that use this method (although that is not the case). Second, why did the author limit the search to the “83 most populous zip codes”? Why not include all zip codes? Does this relate to the number of people participating in TRICARE, or is this because there are simply more people living in those zip codes? Please include a justification here on why there is a population cutoff. Third, on page 8, the author writes that there were 22 states that were included, but the list only included 21 states (from my understanding). In addition, why were some of the zip codes (eg, St. Louis, Rock Island Arsenal) excluded and others included (eg, Amarillo, Lubbock, and El Paso areas only for Texas)?*

Response: I have done my best to expand on the Methods section. I went back and had to comb through all the raw data. In fact, a large number of zip codes contain no humans (just raw land or no population). As western states are less population dense than TRICARE East states, the easiest solution was to use a minimum population as a filter. Unfortunately, I have to run searches manually (rather than via Python) to comply with government regulations. Otherwise, yes, I could just hack TRICARE West and search everything in 5 minutes! The project took over 1 year and resulted in a whistleblower complaint. You can imagine the nightmare.

22 versus 21 states: TRICARE West covers only 21 states. For whatever reason, they decided to include zip codes and a state (containing no providers) from non–TRICARE West regions. To simplify things, I excluded those meaningless data from this revision.

*3. Results: The author generally states that their findings are consistent with past research but do not include a list of articles to which they are referring. Only one article is referenced [* *3* *]. Please provide further support for that claim (in other words, please include all other studies to support the claim of consistent findings). In addition, when discussing results like on page 10, the presentation is difficult to follow with all the zip codes listed and separated by a hyphen. Please consider reorganizing this presentation or placing the list of zip codes in a footnote to ease the presentation of results.*

Response: I added more data on consistency

I added a whole section on significance.

*4. Discussion: The significance of the study could be further elaborated on. At the moment, it pushes for more transparency, but the author could use their data more to discuss the impact of their findings. For example, why would publishing the NPI numbers help patients? What do patients or the author want to gain from that transparency? How can this help future patients or hold physicians more accountable? The discussion loosely taps into the implications, but the study could really tease out this argument more.*

Response: I tried to clarify how/why NPI numbers could help patients. I also tried to clarify the limitations that administrators face as a result of health care labor shortages.

##### Minor Comments

*1. Clean up the grammar and punctuation. For example, on page 4, the author states, “Nicholas et al performed a cross-sectional study of 8204 Medicare beneficiaries who received care from excluded providers. It revealed that patients treated by fraudsters experience a 13%-23% increased risk of mortality and 11%-30% higher risk of hospitalization (Nicholas et al, 2019).” Note, that the start of the sentence, “Nicholas et al” needs a period and a year in the citation.*

*2. I suggest adding a numerical list when discussing the different databases that are available for searching a physician. For example, on page 5, the author lists several different databases starting with the sentence “Multiple public databases exist to search names with respect to each of these issues, including...” Adding in a numbered list can make the information more digestible for the audience. This can also be cleaned up (ie, adding a numeric list) on page 7 when listing the different databases that the physicians were screened in.*

*3. Page 6, it is stated that 203 names appeared in up to 3 additional types of databases. However, what are these 3 additional types of databases? Is it referring to the earlier-mentioned databases? This is unclear.*

Response: I fixed the grammar and other issues.

### Anonymous [4]

#### General Comments

*This paper examines the list of TRICARE providers eligible to deliver telehealth whose names appear on one or more federal sanction lists. This work could have a high impact with implications for national security and patient safety. However, it is not well organized and does not seem to adhere to the scientific method.*

#### Specific Comments

##### Major Comments

*1. This is important work, but is it actually science? A team compared two lists. There are no statistics, minimal numbers, and only one hard conclusion (improper monies). Lots of speculation, but no real answers. Because you are calling out the Defense Health Agency (DHA) and the Military Health System (MHS) on inadequate oversight, your conclusions must be driven by airtight methodology and presented in a professional and well-organized manner. Otherwise, you may just submit this work to the DHA’s OIG as you’ve already done and call it completed.*

Response: I reorganized the content and attempted to obey the scientific method.

*2. Assuming you decide to go with the science, this article has important things to say, but it is not yet ready for publication. It is poorly organized, somewhat informal in tone, and comes across as inflammatory in places. An example of poor organization is the focus on cyber threats and potential in the Introduction, improper monies paid to sanctioned providers in the Results, and a distrust of provider data in the Discussion. Patient safety is not discussed until page 17. Recommend mentioning all these issues in the Introduction and then addressing them in the Results/Discussion in a systematic, organized fashion. Also, streamline areas where the same data is repeated multiple times.*

*3. Similarly, I recommend keeping all the DHA/MHS recommendations together and at the end of the Discussion section, and addressing these in a systematic and organized fashion. “Based on these results, the DHA/MHS/ TRICARE/ whoever should consider the following: (1) Recommendation 1. (2) Recommendation 2...” etc. (Don’t need to take my wording but this is a general idea.)*

Response: I attempted to reduce the inflammatory language, place patient safety stakes up front, and group the core issues in the Introduction.

I also organized the data in a cleaner way using Google Maps to emphasize geographic relationships between the data sets.

Finally, I provided substantially more evidence to support my correlations and fewer opinions.

*4. If there are other people who participated in this study, they should be included as authors or in the Acknowledgments. I doubt that one person compared tens of thousands of names solo.*

Response: Unfortunately, I am a military employee with no grant funding. I conducted all of this work alone over 12 months. I did my best to acknowledge my colleagues, but I had no assistants or other helpers.

## Abbreviations

DHA: Defense Health Agency
LEIE: List of Excluded Individuals and Entities
MHS: Military Health System
NPI: National Provider Identification
OIG: Office of Inspector General
